# Manifestation of SARS-CoV-2 Infections in Mink Related to Host-, Virus- and Farm-Associated Factors, The Netherlands 2020

**DOI:** 10.3390/v14081754

**Published:** 2022-08-11

**Authors:** Wendy J. Wolters, Myrna M. T. de Rooij, Robert Jan Molenaar, Jan de Rond, J. C. M. Vernooij, Paola A. Meijer, Bas B. Oude Munnink, Reina S. Sikkema, Arco N. van der Spek, Marcel A. H. Spierenburg, Renate W. Hakze-van der Honing, Wim H. M. van der Poel, Marion P. G. Koopmans, J. Arjan Stegeman, Lidwien A. M. Smit, Marieke Augustijn-Schretlen, Francisca C. Velkers

**Affiliations:** 1 Department of Population Health Sciences, Faculty of Veterinary Medicine, Utrecht University, 3584 CL Utrecht, The Netherlands; 2 Institute for Risk Assessment Sciences (IRAS), Utrecht University, 3584 CM Utrecht, The Netherlands; 3 Royal GD, 7418 EZ Deventer, The Netherlands; 4 Department of Viroscience, Erasmus MC, 3015 GD Rotterdam, The Netherlands; 5 Netherlands Food and Consumer Product Safety Authority (NVWA), 3511 GG Utrecht, The Netherlands; 6 Wageningen Bioveterinary Research, 8221 RA Lelystad, The Netherlands

**Keywords:** mink, mink farms, SARS-CoV-2, risk factors, disease outbreaks, biosecurity, zoonoses and reverse zoonoses, one health, animal reservoirs, spillover and spillback

## Abstract

SARS-CoV-2 outbreaks on 69 Dutch mink farms in 2020 were studied to identify risk factors for virus introduction and transmission and to improve surveillance and containment measures. Clinical signs, laboratory test results, and epidemiological aspects were investigated, such as the date and reason of suspicion, housing, farm size and distances, human contact structure, biosecurity measures, and presence of wildlife, pets, pests, and manure management. On seven farms, extensive random sampling was performed, and age, coat color, sex, and clinical signs were recorded. Mild to severe respiratory signs and general diseases such as apathy, reduced feed intake, and increased mortality were detected on 62/69 farms. Throat swabs were more likely to result in virus detection than rectal swabs. Clinical signs differed between virus clusters and were more severe for dark-colored mink, males, and animals infected later during the year. Geographical clustering was found for one virus cluster. Shared personnel could explain some cases, but other transmission routes explaining farm-to-farm spread were not elucidated. An early warning surveillance system, strict biosecurity measures, and a (temporary) ban on mink farming and vaccinating animals and humans can contribute to reducing the risks of the virus spreading and acquisition of potential mutations relevant to human and animal health.

## 1. Introduction

Since December 2019, SARS-CoV-2 has spread rapidly all over the world, causing respiratory disease in humans, with a varying degree of severity ranging from a mild infection to death [1,2,3,4,5,6]. Furthermore, many different animal species in households (cat, dog, and ferret), on fur farms (mink), in zoos (including lion, tiger, puma, snow leopard, gorilla, hippopotamus, manatees, and otter), wildlife (including white-tailed deer, feral mink and otter) and after experimental infection (including rabbit, several rodent species, bats, monkeys, macaques, marmoset, skunk, tree shrew, raccoon dog, raccoon, white-tailed deer) have shown to be susceptible to SARS-CoV-2 [3,7,8,9,10,11,12,13,14].

At the beginning of 2020, the Netherlands counted 126 mink farms, of which 69 were confirmed to be infected with SARS-CoV-2 between the end of April to the end of November 2020 [15]. Furthermore, in the US, Canada, and several European countries, mink farms were infected [12,14,16,17,18,19,20,21,22,23]. The intended ban on mink farming in the Netherlands was brought forward from 2024 to early January 2021, because of concerns for mink to become SARS-CoV-2 virus reservoirs, especially also in light of virus evolution. Furthermore, several other countries, including Denmark, implemented a complete or temporary ban on mink farming [7]. Mink farming continues in other European countries [7] and also in the US, Canada, Russia, and Asian countries [21,24]. Consequently, for these countries, the potential (transboundary) risk to animal and human health remains.

In order to take appropriate measures to reduce risks associated with these virus infections on mink farms in the future, it is imperative to increase the knowledge about possible risk factors for virus introduction, including factors related to susceptibility to infection and transmission between mink and mink farms. Earlier studies have indicated associations between SARS-CoV-2 infections on mink farms with mink age [17], farm distances [15,17], farm size [17], and infected personnel [15]. Host-associated factors related to susceptibility to several diseases in farmed mink have been described previously [25,26,27,28] and included sex, age and coat color. The latter can be explained by coat color being related to other genotypic traits, including genetic factors related to the immune system [29,30,31]. However, comprehensive insight into clinical signs and potentially associated risk factors remains limited, despite the available data on SARS-CoV-2 infections in farmed mink to date [12,14,16,17,19,20,22].

Increasing knowledge of factors related to the susceptibility of mink to SARS-CoV-2 infections and clinical manifestation of these infections is important to facilitate early diagnosis and hence fast action. This will reduce the risks of a further spread between mink and people and to other mink farms, which has been shown to be a major problem in multiple countries [7,21]. In addition, studying infections on mink farms with many animals kept together of young and adult age can also yield important generic insights about the behavior of SARS-CoV-2 in populations. Furthermore, the clinical and diagnostic representation of SARS-CoV-2 infections on mink farms may also increase insights that are relevant for these and similar infections in domestic, farmed, and wild animals, as well as humans, in view of the One Health context.

Therefore we performed this comprehensive study, including detailed data collection and laboratory tests involving all SARS-CoV-2 affected mink farms in the Netherlands. The objectives of this study were to (i) systematically assess and describe farm- and outbreak characteristics; and (ii) study associations with host-, virus- and farm-related factors.

## 2. Materials and Methods

### 2.1. Study Population

This study includes the data from all 69 Dutch mink farms diagnosed with SARS-CoV-2 infections in the Netherlands. The first farm was diagnosed on 24 April 2020, and the last farm on 4 November 2020. After pelting of the remaining farms, mink farming was discontinued in the Netherlands from January 2021 onwards. Data from non-infected farms were not analyzed for this study. The farms are referred to by a farm-ID consisting of NB (abbreviation for Dutch word for mink farm) followed by a consecutive number according to the time of diagnosis. The first 2 farms were named NB1a and NB1b, followed by NB2-NB68.

Mink farming follows an annual production cycle which starts with mating in March, whelping in April and May, followed by weaning of the kits in June and July. The kits are then raised until the end of the year, when in November and December, most minks are euthanized for pelting, and a selection is kept as breeding stock for the next year. Further details on mink farming practices in the Netherlands, surveillance and control measures, and the course of the SARS-CoV-2 outbreaks on mink farms have been described previously [7,15,32,33].

### 2.2. Sample and Data Collection on All 69 Infected Mink Farms

Mink farms were suspected based on (i) mandatory notification of clinical signs; (ii) a positive result of an RT-PCR against the SARS-CoV-2 E gene [34] from early warning surveillance (EWS), which included weekly mandatory screening of recently dead mink on all farms; or (iii) a positive result during mandatory serological screenings with an in-house indirect IgG ELISA at Royal GD (GD, Deventer, the Netherlands) [35]. The 1st serological screening was performed in May and June, and the 2nd in September 2020 on all remaining mink farms. All mink farms suspected to be infected were visited for clinical inspection and official confirmational sampling with 20 throat and rectal swabs taken by a team of veterinarians of the competent authority, i.e., the Netherlands Food and Consumer Product Safety Authority (NVWA, Utrecht, the Netherlands) and GD, and were tested with RT-PCR at the national reference laboratory (Wageningen Bioveterinary Research, Lelystad, the Netherlands). The rectal and throat swabs were taken from minks with clinical signs or from mink in cages with prior observed mortality if present, and otherwise randomly. The virus cluster on farm level, based on sequencing of positive mink samples, was determined at Erasmus MC (Rotterdam, the Netherlands), as described previously [15,36,37].

The NVWA performed investigations on all 69 SARS-CoV-2 infected mink farms. This included an interview with the farmer to record information on the outbreak and animal and farm characteristics. All files containing the details of this investigation, including contact tracing, history of clinical signs, type of housing, number of housed mink, biosecurity, date, and reason of suspicion, were analyzed in this epidemiological study. In addition, an in-depth survey was performed on 31/69 farms that were willing to participate and had not yet been included in a previous study [11]. The survey consisted of questions related to potential introduction and transmission routes and risk factors, including people, vehicles, feed, materials, animals (pets, farm animals, feral cats, rodents, insects, and wildlife, such as birds, wild carnivores, or bats) on the farm premises. Furthermore, the history of clinical signs and further farm information, such as a map of the farm and details on housing and ventilation were included, but not for all farms were answers to all questions obtained. For farms that did not participate in the surveys or for which available data were limited, additional information was retrieved by interviewing veterinarians that visited the farm for sampling or the farm veterinarian.

### 2.3. Additional Extensive Sample and Data Collection on Seven of the Infected Mink Farms

On 7/69 farms, additional extensive random sampling of mink was performed a day before or on the day of culling. The reason for this was that some farms (15/69) tested negative at official sampling of 20 mink after notification of clinical suspicion or a positive EWS test result but tested SARS-CoV-2 positive a few weeks later. To rule out that SARS-CoV-2 had circulated on farms for several weeks before detection, in case of low initial infection prevalence, seven farms, i.e., NB46, 48, 50, 52, 53, 54, 61, were selected for this additional sampling. These farms had been previously suspected and were eventually diagnosed between 3 September and 2 October 2020 and were all diagnosed with the same virus cluster (i.e., cluster A), as described previously [15].

Based on a map of sheds, rows of cages, total animal numbers, and distribution of the minks (kits, adult males, adult females, and different coat colors) on the farm, a preselection was made of the number of animals to sample in the different parts of the farm to ensure complete coverage of the entire farm and inclusion of all sexes, age groups and mink types. Throat swabs and blood samples (on filter paper after nail clipping) were collected, and the location on the farm, type of animal (i.e., age, sex, and coat color), and whether or not clinical signs were present was recorded for each sampled mink. Presence of viral RNA (positive with Ct-value < 35), Ct-values from the RT-PCR (for samples with Ct-value < 35) as a measure of viral load, and the presence of antibodies (with the indirect ELISA as described at Section 2.2) were analyzed. Throat swabs taken at NB46 and NB48 were excluded from the statistical analyses as swabs were pooled for the laboratory analyses, and test results could thus not be linked to individual animals.

### 2.4. Data Analysis

All analyses were performed with software package R studio version 1.3.1093 [38].

#### 2.4.1. Clinical and Diagnostic Data Analyses for All 69 Infected Mink Farms

A list of 19 possible clinical symptoms (Table S1), categorized into general signs, respiratory signs (divided into signs of the upper or lower respiratory tract), and signs of the gastrointestinal tract, was used to score all reported clinical signs on farm level for the different mink categories (kits, females or unknown) from the available data for each infected mink farm. The available data included the clinical signs scored on a standardized form filled out by the NVWA veterinarian during the official clinical inspection and sampling, which was available for all 69 farms. Additionally, further details on the observed clinical signs, obtained from surveys of the farmer and interviews of NVWA, GD, and the farm veterinarians, were used to further complete the dataset.

The clinical and diagnostic data of all 69 farms were first inspected and visualized using descriptive analyses. Subsequently, the association of total number of clinical signs (from the total of 19 different clinical signs) observed at the farm was assessed by a clustered univariable grouped binomial logistic regression model (glmer, library lme4 [39]). The respective explanatory variables were: virus cluster (A, C, and D, as described in Lu et al., 2021 [15]), percentage of PCR-positive throat swabs from the 20 sampled minks (in three categories, i.e., <25%, 26–74% and >75% positive), type of housing (sheds, halls or both), farm size (small: <30,000; medium: 30–50,000; large: >50,000), and date of diagnosis (before 1 August or from 1 August onwards). Virus clusters B and E were both only detected at one infected mink farm and therefore excluded from the statistical analyses. The cutoff values for the percentage of positive swabs and farm size were chosen to create an equal distribution of the data over the categories. For the date of diagnosis, the two categories were based on the observation that from August (NB27) onwards, an increase was seen in the percentage of positively tested mink and the number of clinical symptoms observed on the newly diagnosed farms (Table S2). A random effect for farm was added to the model for the clustered number of symptoms out of 19. Associations between the total number of clinical signs and the tested variables are presented as unadjusted odds ratios with 95% confidence interval (95% CI).

Differences in the total number of observed clinical signs (from the total list of 19 potential signs) for the different farms between adult females and kits were analyzed using an independent sampled *t*-test. The Chi-square test of independence was used to study associations between the categorized percentage of positive throat swabs and the independent variables: virus cluster, type of housing, farm size, and date of diagnosis (using the same categories as described above) and to compare the presence of clinical signs (detected at time of official sampling vs. the absence of visible clinical signs) on the different farms between kits and females. The level of significance was set at α = 0.05.

#### 2.4.2. Clinical and Diagnostic Data Analyses for the Seven Extensively Sampled Mink Farms

Mixed effect logistic regression models were used to study associations between the presence of clinical signs (present vs. absent) in an animal and the variables coat color, age, sex, and ELISA results. Age and sex were combined into the categories: juvenile males, juvenile females (both <1 year of age), and adult females (>1 year of age). Adult males were only present in very low numbers and not on all farms and were therefore excluded from the statistical analyses. As differences in disease susceptibility have been described especially for dark compared to light-colored mink, coat colors were categorized as dark (wild, brown, or mahogany (MAH)), light (silver blue (SBL), and silver cross (SCR)) or other coat colors (jaguar, pearl, black, other). A random effect for farm was added to the model to take scoring of several mink within the same farm into account. Based on the lowest Akaike’s information criterion (AIC) using stepwise backward single term deletions, noncontributing factors to the model were removed to choose the best fitting model. The results of the best fitting model are presented as adjusted odds ratios and 95% CI.

As Ct-values could be censored (no exact Ct-value for negative samples with Ct > 35), we used cox proportional hazard analysis to analyze associations between Ct-values and coat color (dark, light, or other coat colors), the combination of age and sex (juvenile males, juvenile females and adult females), and the presence of clinical signs (present vs. absent) as explanatory variables using the functions survfit and coxph. Farm was added as random effect to take the correlation between sampled animals within a farm into account. Of the model with significant associations between Ct-values and the tested variables, the results were evaluated based on hazard ratios and 95% CI.

## 3. Results

Data on all 69 mink farms diagnosed with SARS-CoV-2 infections were obtained. For these farms, information on general characteristics (Section 3.1) and the clinical and diagnostic representation (Section 3.2) was obtained, but data were incomplete for some of these farms. On a subset of seven mink farms diagnosed in September and October (NB46, 48, 50, 52, 53, 54, and 61), additional random sampling throughout the farm was performed, which resulted in clinical data from 130, 155, 112, 232, 314, 267 and 193 animals from different age groups and sex respectively (Section 3.3).

### 3.1. Farm Characteristics

#### 3.1.1. Type, Size, and Location of Farms

The mink were housed in wire netting cages placed in open sheds (39 farms), closed halls (21 farms), or both (6 farms), or the housing type was unknown (3 farms). The numbers of minks per farm were highly variable, ranging from 2,050 to almost 100,000 (Table 1). No significant associations were found between the clinical (i.e., type and number of different clinical signs) or the percentage of positive swabs with the different housing types or farm sizes.

All 69 infected farms were located in the southeastern part of the Netherlands in the province of Noord Brabant (44), Limburg (23), and Gelderland (2) [15]. This area was densely populated with mink farms. Results showed that 74% of the infected mink farms (51/69) were located within three km of another infected mink farm and 65% of the infected mink farms (43/66) within three km of another infected farm with the same virus cluster (Table 1). Neighboring farms, in some cases, had a different virus cluster; however, from August onwards, stronger geographical clustering was seen mainly concerning cluster A as described previously by Lu et al. (2021) [15] and Velkers et al. (2021) [37].

#### 3.1.2. Humans on the Farm and Hygiene Protocols

Certain farmers owned multiple infected mink farms (32/69) (Table 1). The farms were mainly visited by the owners, permanent and seasonal employees, and sometimes by family members. On 35/69 farms, a maximum of five people were reported entering the sheds or halls. No more than two permanent employees were employed on 44/69 infected farms and 54/69 farms had no more than three seasonal employees, which often returned to the same farm every year or lived nearby or on the farm premises. Temporary employees were hired on 18/69 farms. Furthermore, 36/65 farms were known to exchange employees once or more with one or more other farms. For 31/51 farms, it was known that employees showed SARS-CoV-2-related signs (Table 1).

Hygiene measures were implemented by the Dutch Ministry of Agriculture, Nature and Food Quality (LNV) and the Ministry of Public Health, Welfare and Sport (VWS). After the first two outbreaks in April, mink farm owners and their veterinarians were obligated to notify respiratory signs and increased mortality in mink. From 19 May 2020 onwards, SARS-CoV-2 was officially assigned as a notifiable infectious animal disease, and strict biosecurity and biocontainment measures were implemented on all mink farms, including a ban on mink and manure transport, visitor restrictions, and obligatory use of personal protective equipment (PPE) for all staff and visitors of mink farms. Furthermore, mink farmers were asked to prevent any contact between their mink and other animals and to keep cats, dogs, and other animals on their farms. From June 2020 onwards, the government decided to cull all mink of infected farms, and from July 2020 onwards, following increasing indications of SARS-CoV-2 infections of employees, medical mouth masks became mandatory on farms suspected of infection and during culling, or when one of the workers tested SARS-CoV-2 positive. In September 2020, employees of infected mink farms were advised to get tested, and a 10-day waiting period for the exchange of personnel between farms was implemented. For pelting on non-infected farms, additional hygiene measures, including using FFP2 mouth masks, were implemented [33,37].

According to the farmers, following the measures consistently all the time proved to be difficult in practice. Non-medical face masks were often used, but additional measures, such as splash goggles or medical face masks, were hardly used or by a limited number of people at the farm. The farmers experienced problems with the combination of splash goggles and face masks, as this resulted in fogging of the goggles. The use of medical face masks was obligatory solely during culling and pelting.

Although 6/63 farms were known for sharing materials and/or vehicles with other mink farms (Table 1), based on the timing of infection and virus sequence analyses, this was not the route of transmission of SARS-CoV-2 between these farms.

#### 3.1.3. Veterinary and Feed Service Contacts and Manure Management

Many farms shared the same feed supplier, and many also the same factory. Mink feed was delivered almost daily into a feed silo. On most farms, the silo was accessible from the main road, and in case the vehicle needed to enter the farm yard, appropriate hygiene protocols were followed. The same vehicle delivered feed subsequently to multiple other farms, but infections of mink farms were not linked to the same vehicle or feed factories. Furthermore, a total of 29 samples of complete rations from five major feed suppliers were PCR tested in June 2020 for another study and were SARS-CoV-2 negative [40].

A total of six veterinary practitioners visited mink farms, two of whom worked for the same company and visited the majority of the farms (54/69). Contact tracing and virus sequencing results did not suggest any relationship between the farm visits by veterinary practitioners and outbreaks on different farms.

For 21/60 farms, it was known that stored slurry was deposited onto pastures, usually near their own farm, in the period before the farm was diagnosed with SARS-CoV-2 and manure application became prohibited (Table 1). The pastures on which the slurry was applied were often also located near other mink farms (considering the overall high density of mink farms in this area) but based on the time and location of application of the slurry, and time of diagnosis and location of newly infected farms, a relation was not evident.

#### 3.1.4. Other Animals

Dogs were present at 41/66 and domestic cats at 9/60 farms (Table 1). For some of the farms, it was likely that the dogs and domestic or feral cats could make physical contact or indirect contact with the mink through feed, straw, and other materials. This may have caused SARS-CoV-2 infections in cats and dogs on some of these farms, as described previously [13]. The majority of the farmers (50/67) reported feral or neighboring cats on or in the area around the farm. Additionally, 27/49 of farms housed other animals, including cattle, sheep or goats, deer, cows, horses, pigs, poultry, rodents, turtles, foxes, and wallabies (Table 1).

A few farms reported visits by carnivores such as red foxes (*Vulpes vulpes*) (14/54), badgers (*Meles meles*) (8/54), and mustelids (14/54) such as weasels (*Mustela nivalis*), stone martens (*Martes foina*), American mink (*Neovison vison*) and polecats (*Mustela putorius*) at or in the area around the farm. Bats were seen by the mink farmers at 13/54 farms but not often inside the sheds or halls [37,40]. The majority of the farms reported bird species which were mainly corvids, including crows (*Corvus corone*) and jackdaws (*Corvus monedula*) (28/50), sparrows (*Passer domesticus*) (24/50), and starlings (*Sturnus vulgaris*) (13/50). Other bird species reported were pigeons (*family Columbidae*), magpies (*Pica hudsonia*), gulls (*family Laridae*), blackbird (*Turdus merula*), robin (*Erithacus rebucelablba*), coal tits (*Parus major*), barn swallows (*Hirundo rustica*), finches (*family Fringillidae*), white wagtails (*Motacilla alba*), owls (*order Strigiformes*) and common buzzards (*Buteo buteo*). Birds were seen in feed and straw storages, both in and on top of sheds and halls and even on top of and in the animal cages [41].

Despite pest control, the presence of mice and rats was reported at 18/50 mink farms. Farmers of 21/24 farms complained about insects such as flies, wasps, and beetles (Table 1).

### 3.2. Clinical and Diagnostic Representation of SARS-CoV-2 in Mink

#### 3.2.1. Analyses of Clinical Signs Observed on All Farms in Kits and Adult Mink

**Figure 1:**
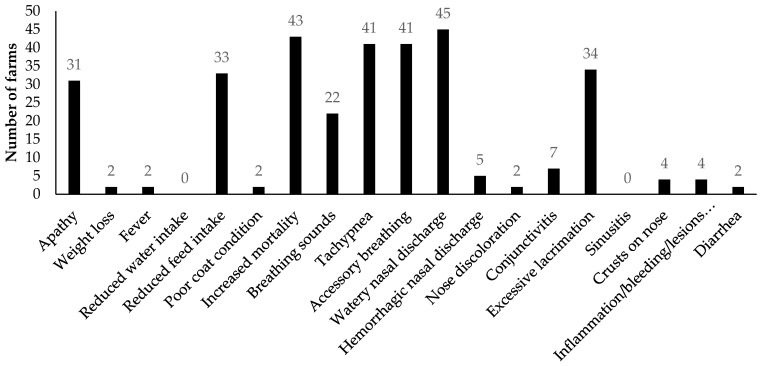
Overview showing per clinical sign, of the 19 different clinical signs of the standardized list (Table S1), the number of farms (*n* = 62) for which the sign was observed in kits, male or female adult minks. Note that inflammation/bleeding/lesions refer to these on the gingiva.

**Figure 2:**
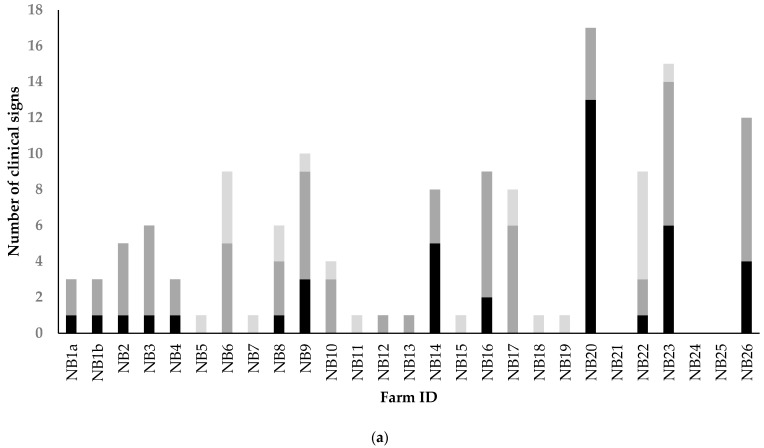

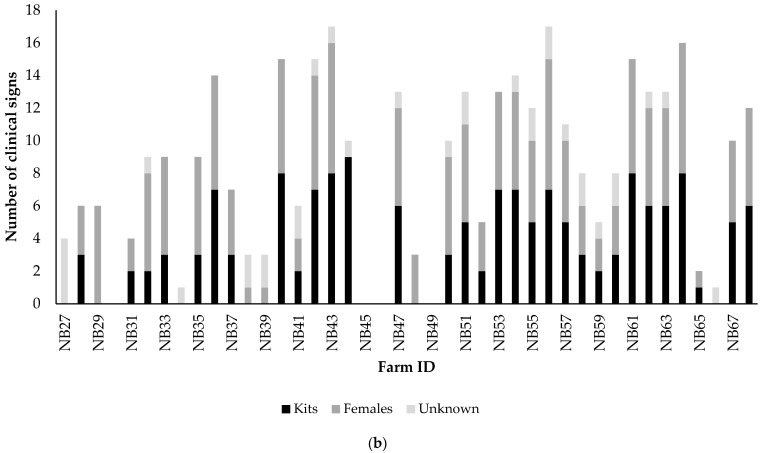
The total number of clinical signs observed on the SARS-CoV-2 infected mink farms in kits, female adults or in minks of unknown sex or age category, in case this information could not be found in the available records. The X-axis shows the farm-ID which consists of NB (abbreviation for Dutch word for mink farm) followed by a consecutive number according to the time of diagnosis. (**a**) NB1a-NB26, diagnosed between 31 May and 1 August; (**b**) NB27-NB68 diagnosed between 1 August and 4 November 2020.

In total, 62 of the infected mink farms reported clinical signs, which were mainly signs attributed to the respiratory tract and signs of general disease such as apathy, reduced feed intake, and increased mortality (Figure 1 and Figure 2). Respiratory signs frequently reported were watery nasal discharge, tachypnea, accessory breathing, excessive lacrimation, and breathing sounds like coughing and sneezing. Signs of the gastrointestinal tract, i.e., diarrhea and bleeding, inflammation, or lesions of the gingiva, were hardly reported (Figure 1). Kits showed a more variable clinical representation as more different signs were observed in kits than in adult females (Figure 2). More details on the clinical and pathological representation of SARS-CoV-2 infections on NB1-4 were described previously by Oreshkova et al. (2020) [11] and Molenaar et al. (2020) [42].

No significant differences were found in the total number of clinical signs between kits and adult females. In contrast, the proportion of farms where clinical signs were observed in adult females was significantly higher compared to the proportion of farms with clinical signs observed in kits (0.75 vs. 0.64; Chi-square, X-Squared = 26.41, *p* < 0.05) (Table S3). From August onwards, more often and more different signs were observed (Figure 2).

The mean number of clinical symptoms observed in kits increased on farms diagnosed from August onwards, and the odds of observing a symptom in kits were 4.25 times higher from August onwards compared to before August (Table 2). In adult females, a slight increase in the mean number of clinical symptoms was seen, but the odds of observing clinical signs in adult females were not significantly higher from August onwards (Table 2).

Phylogenetic analysis of the mink SARS-CoV-2 genomes showed that sequences of all farms were grouped into five different clusters; clusters A (41), B (1), C (15), D (7), and E (1) [15,32]. At four mink farms, the cluster could not be determined (Table 1). In mink farms infected with virus clusters A and C, more clinical signs were reported compared to farms infected with cluster D (Table 2). The odds of observing a clinical symptom for cluster D farms were significantly lower compared to cluster A and cluster C farms (Table 2). There was no difference in the type of signs for the different clusters.

No significant differences were found in the number of different clinical signs (of the 19 clinical signs) for the different types of housing (sheds, halls, or both), different categories of PCR test outcomes (<25%, 26–74%, and >75% positive) and farm size (small: <30,000; medium: 30–50,000; large: >50,000).

#### 3.2.2. Analyses of Diagnostic Results on All Farms

**Figure 3:**
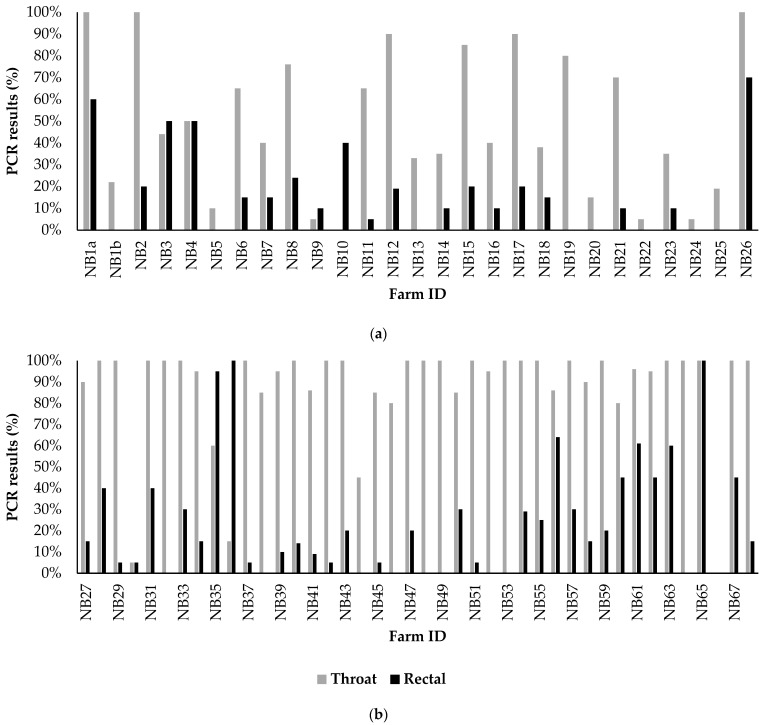
Percentage of SARS-CoV-2 positive rectal or throats swabs taken at official sampling by the competent authority. Twenty rectal and 20 throat swabs were taken from minks with clinical signs or from mink in cages with prior observed mortality if present, and otherwise randomly. (**a**) NB1a-NB26, diagnosed between 31 May and 1 August; (**b**) NB27-NB68 diagnosed between 1 August and 4 November 2020.

Comparisons between SARS-CoV-2 RT-PCR results of the throat and rectal swabs showed that throat swabs had the highest sensitivity (Figure 3). The majority (45) of the farms tested positive on throat swabs, with over 75% positive of the 20 swabs taken at official sampling, while most of the rectal swabs tested negative or less than 25% tested PCR positive. A total of 17 infected farms tested negative with the use of rectal swabs, while those same farms tested positive with the use of throat swabs (Table S2).

Similar to the increase in clinical signs during the course of the epidemic, the percentage of positively tested throat swabs also increased (Figure 3, Tables S2 and S4). Throat swabs tested more positive, often close to 100%, for farms diagnosed from August onwards compared to farms diagnosed before August (Chi-square, X-Squared = 24.6, *p* < 0.05). No significant differences were found in the number of signs between the different categories of PCR test outcomes (<25%, 26–74%, and >75% positive). Furthermore, no significant differences in PCR test results of the throat and rectal swabs were found for the different virus clusters.

### 3.3. Results of Analyses of Extensive Random Sampling on Seven Mink Farms

All tested (pools of) throat swabs from the seven farms were PCR positive, whereas only 144/1523 mink showed clinical signs (Table S5). Mink with coat colors wild, brown, or mahogany were more often clinically affected compared to the colors silver blue and silver cross (12% vs. 4%) (Table 3). In addition, clinical signs were significantly more often observed in juvenile males (11%) and in adult females (9%) compared to juvenile females (5%) (Table 3). The odds for detecting clinical signs were 2.64 higher in ELISA positive compared to ELISA negative animals (Table 3).

Ct-values in PCR-positive throat swabs from juvenile males were, on average, significantly lower compared to juvenile females (mean Ct-value 21.26 vs. 22.36), indicating a higher viral load for juvenile males (Table S6). Furthermore, adult females showed higher Ct-values compared to juvenile males (21.26 vs. 22.34). No significant associations were found between Ct-values and the different coat colors or the presence of clinical signs.

## 4. Discussion

This study provides an overview of the farm- and outbreak characteristics and clinical and diagnostic data for SARS-CoV-2 outbreaks on Dutch mink farms in 2020. Moreover, associations between the manifestation of SARS-CoV-2 infections in mink in relation to host-, virus- and farm-associated factors were evaluated. We aimed to contribute to knowledge into possible risk factors for virus introduction, including factors related to susceptibility for infection and transmission between mink and mink farms, which may help reduce risks of SARS-CoV-2 infections, and potentially provide insights for similar zoonotic or pandemic pathogens for future One Health challenges.

In previous studies, an impressive amount of knowledge has been gathered on host-pathogen interactions for SARS-CoV-2. The host cell receptor used for viral entry, i.e., the angiotensin-converting enzyme 2 (ACE2) that interacts with the receptor-binding protein (RBD) on the Spike glycoprotein of SARS-CoV-2, is expressed on the surface of many cell types, including pulmonary, renal, cardiac, intestinal, and endothelial cells [43]. SARS-CoV-2 can infect many mammalian species due to the conservation of ACE2 across mammals. Moreover, it transmits efficiently in several host populations [44]. Variations in the ACE2 amino acid sequence can significantly impact host susceptibility, and multiple factors, including age, cell type, species, and genetic polymorphisms, can determine the outcome of ACE2 interactions [43]. For SARS-CoV-2 in mink, extensive analyses of Spike protein mutations and their effects on the 3D structure and binding capacity and how this may impact epitope targets for the immune response have been described [43,45]. Such studies on RBD and ACE2 interactions for different virus lineages and host species facilitate predictions of host susceptibility and potential for cross-species transmission [43,46,47,48,49,50]. However, in vitro and in silico receptor binding potential is not necessarily associated with successful replication or disease in vivo [46]. Consequently, in vivo studies and descriptions of actual field cases, as described in this paper, remain essential elements for studying infectious diseases with zoonotic and pandemic potential.

On the majority of Dutch mink farms (62/69), mild to severe respiratory signs, ranging from watery nasal discharge to severe respiratory distress and interstitial pneumonia, and general diseases such as apathy, reduced feed intake, and increased mortality were detected [11,42]. On less than 3% of the farms gastrointestinal signs, such as diarrhea and inflammation, bleedings and lesions in the gingiva were found. These findings are consistent with previous reports of symptoms of SARS-CoV-2 infections of farmed mink in several countries, where especially signs of the respiratory tract, slightly increased mortality, and in some cases (rarely) gastrointestinal signs were reported [7,12,17,19,20]. Moreover, the observed clinical signs can also be explained by the ACE2 distribution in mink. ACE2 shows abundant and widespread presence in the upper respiratory tract, especially in the nasal turbinates [51] in ferrets and minks. This corresponds with the presence of upper respiratory tract infection in both species and suggests high susceptibility to infection via respiratory droplets or aerosols, which can facilitate virus transmission. The presence of ACE2 in the lower respiratory tract, only in mink, is also consistent with lower respiratory tract disease as seen in mink, but not in ferrets [51].

The predominance of clinical signs in the respiratory tract was also in line with the diagnostic outcomes. The (pooled or individually tested) throat swabs in the seven extensively sampled farms were all PCR positive with low Ct-values, indicating a high prevalence and high viral load. A total of 17 infected farms tested SARS-CoV-2 negative in rectal swabs, while throat swabs were positive. This indicates that, on average, 27% of the infected farms could have been missed if only rectal swabs had been taken. This difference in sensitivity between the throat and rectal swabs further supports that SARS-CoV-2 replicates efficiently in the respiratory tract of minks, which was also shown in experimental studies with mink [52,53]. Furthermore, in cats, rectal swabs tested negative while throat swabs tested positive for SARS-CoV-2 [13], suggesting that throat swabs are preferable for testing domestic and farmed animals. Nevertheless, virological surveillance using rectal swabs in animals does have diagnostic value. ACE2 is present in the brush border of small intestinal enterocytes of mink, cats, and most other species. Therefore, productive infections of the gastrointestinal tract are likely to occur in animals, resulting in positive rectal swabs. Additionally, positive rectal swabs may be a result of oral ingestion of virus particles from mucus from the respiratory tract or by the grooming of other animals when housed together [51]. Strikingly, although 100% of the tested mink on extensively sampled farms was PCR positive, only 9.4% of the sampled minks showed clinical signs. This indicates that the surveillance system used in the Netherlands, with in addition to obligatory notification of clinical signs a mandatory early warning surveillance with frequent testing of dead mink (by means of throat swabs), and serological screenings, has been imperative for the early detection of infected farms.

Clinical signs differed between virus clusters and were more severe for animals infected later during the year and in male and dark-colored mink. The latter is in line with the genetic predilection for dark-colored mink to be more susceptible to epizootic catarrhal gastroenteritis, caused by a coronavirus infection, compared to light-colored mink [28]. In contrast, Aleutian disease (AD) progresses more slowly, and recovery is quicker in dark-colored mink, whereas light-colored mink are more susceptible because they are homozygous for the Aleutian gene [25]. Whether the difference in the susceptibility to SARS-CoV-2 for the different coat colors found in this study was related to a genetic predilection is not clear. Moreover, not all coat colors were present on the farms, and the numbers of mink per coat color were very limited in our dataset, making it difficult to draw generic conclusions on coat colors and SARS-CoV-2 susceptibility.

In our study, we found that male mink were more often and more severely affected compared to females. Furthermore, in farmed minks in Greece, a higher viral load was found in organs from dead male mink compared to female mink [19]. Moreover, in France, it was found that male dogs were more often seropositive for antibodies against SARS-CoV-2 compared to female dogs [54]. This difference may also be related to the high expression of ACE2 in the testes, which may result in higher viral loads, more viral shedding, and a higher risk of becoming symptomatic [55,56].

On infected mink farms in Denmark, clinical signs were reported more often among kits (53%) compared to adults (60%) [17]. On mink farms in our study, clinical signs were initially more often reported in adults compared to kits. However, as the epidemic progressed in time and the kits aged, the odds of observing clinical signs increased in kits on the newly discovered infected mink farms, and also more different clinical signs were seen, whereas, in adults, the reporting of clinical signs remained more or less the same. This may be because it is easier to assess clinical signs in older kits than in young kits, simply because they are bigger and more visible outside the nesting box. Furthermore, with a similar (seasonal) farm cycle between mink farms, kit age was highly correlated with time of diagnosis, with an age of 12 weeks from August onwards. This makes it difficult to distinguish age effects from other time-related factors. For instance, with the increasing age of the minks, also the quantity of feces increases rapidly, and moreover, dustiness increases. This may have promoted the transmission of the virus through the farm. Furthermore, minks start shedding fur in August, which may also make the animals more susceptible to infections [57,58] and may also increase the airborne spread of virus particles emanating from contaminated fur. Moreover, at the start of the epidemic, five different virus clusters were detected [15], of which clusters A and C caused significantly more clinical signs compared to cluster D. Cluster A dominated the outbreaks from September onwards, which may also explain the increase in observed clinical signs later in the epidemic. Nevertheless, age-dependent effects may also have played a role. In children, the ACE2 receptor shows a reduced binding affinity and expression compared to adults, which reduces viral entry [43]. In hamster experiments, animals infected at an older age were more severely clinically affected and showed a more pronounced influx of immune cells in lung tissue [59]. Moreover, a better immune response was found in young mice with less virus replication and milder clinical manifestations compared to aged mice, which is similar to what has been observed in human infections [60].

Sequencing has shown that both human-to-mink and mink-to-human transmission occurred [15,32]. In total, 102 people connected to 42 infected farms tested positive for SARS-CoV-2, and in all cases where sequencing could be performed, the isolated virus in farm workers/families was of the same type as found circulating in the minks on the respective farm [15,37]. Similarities in virus sequences obtained from infected farmers or employees with that from mink from the concerning farm were not only found in the Netherlands [15,32] but in other countries as well [12,16,19].

The emergence of mink-associated SARS-CoV-2 variants with bidirectional transmission between mink and humans raised substantial concerns [24]. Circulation of SARS-CoV-2 in farms with high mink density was feared to increase the risk of mutations, followed by back-jumping to humans of viral lineages with potentially enhanced viral fitness or transmissibility or impacts on vaccine efficacy [44,45]. One of the mutations (i.e., Y453F) found in SARS-CoV-2 variants in mink in Denmark and the Netherlands was shown to affect the RBD in the SARS-CoV-2 Spike protein. This mutation showed a better affinity for ACE2, and some evidence of partial immune escape [24,44,45,50,61,62], which prompted the culling of infected mink farms and (temporary) bans on mink farming in Denmark, the Netherlands, and some other countries. Although the Y453F variants resisted neutralization to an RBD-binding monoclonal antibody [61,62], further studies showed little evidence of potential effects on binding of most other SARS-CoV-2 neutralizing antibodies [45]. Moreover, Y453F variants have shown minimal or no evolutionary advantage for transmission in humans and have remained at low frequencies in the human population [44]. To date, SARS-CoV-2 circulation in mink populations has required minimal virus adaptation, did not increase mutation rates, nor showed advantages for circulation or drastic changes to the SARS-CoV-2 genomic landscape; the same applies for SARS-CoV-2 circulation in white-tailed deer populations [44]. Nevertheless, genomic surveillance of SARS-CoV-2 in human and animal populations remains important to study virus adaptation and potential consequences for human and animal hosts. Furthermore, the low mutational prerequisite for efficient SARS-CoV-2 transmission in novel hosts highlights the ‘generalist’ nature of SARS-CoV-2 as a mammalian pathogen, which suggests that spillover- and spillback events across humans and animals are inevitable and will remain a challenge for control of the virus [44].

Despite strict biosecurity measures issued by the Dutch government, SARS-CoV-2 kept spreading between mink farms in the Netherlands, similar to what was observed in other mink farming countries [12]. Direct contact between infected humans and mink was one of the introduction routes of the SARS-CoV-2 virus into farms. However, only in a limited number of farms could transmission be linked to shared personnel [15,37]. Until the end of July, a lot of human–mink contact occurred during weaning and vaccination, and extra staff was used while visitor registration was not yet required. However, also after the biosecurity measures were intensified, exchange of personnel was not allowed anymore, and direct contact with minks was reduced because of the growing period of the minks, infections kept spreading, and often, people were not diagnosed until after the minks became infected, suggesting that other transmission routes played a role. Furthermore, a strong spatial clustering was found later in the epidemic, but despite extensive analyses, other modes of transmission explaining farm-to-farm spread were not elucidated in the Netherlands nor in other countries [7,15,37].

Implementation and strict compliance to biosecurity measures, such as consistently wearing medical face masks and splash goggles, have been reported to reduce risks of infections in humans [19,63]. This was also illustrated in the Dutch outbreaks by the relatively low number (3/75) of SARS-CoV-2 positively tested workers (unconfirmed to be of a mink variant, as no sequencing data are available) from the competent authorities, who had short but very intensive contact with infected minks during diagnostic visits and culling but adhered to very strict hygiene protocols [37]. These protocols included consistently wearing medical facemasks and splash goggles in addition to protective clothing and footwear. A study by De Rooij et al. (2021) [64] also showed the high risk of exposure to SARS-CoV-2 in infected mink farms after detecting high levels of viral RNA in airborne inhalable dust, which makes it more imperative to follow strict hygiene and biosecurity measures and reduce the risk of becoming infected. However, strict and continuous compliance to biosecurity measures is especially difficult for owners and their employees, which may have limited access or means to use adequate protective gear, have to work on the farm every day, and are less trained or educated in biosecurity than workers from the competent authorities. Therefore, in addition to the provision of personal protective equipment to farm workers, this may need to be accompanied by biosecurity training to ensure its proper and consistent use.

In Denmark, an association was found between farm size and the risk of infection, indicating increased risk with increasing farm size [17]. However, an association with farm size was not found in our study. The proximity to another infected farm may have been a common risk factor for infection in both the Netherlands and Denmark [15,17]. Although for 35% of the neighboring farms, a different virus cluster was detected, from August onwards, stronger geographical clustering was seen mainly concerning cluster A. This has been described in more detail previously by Lu et al. (2021) [15]. To date, it has remained unclear which underlying factors could explain this phenomenon observed in the Netherlands and also other countries [12,17,37]. Strong geographical clustering of infected farms has also been described for other infectious diseases in farm animals, such as bluetongue virus serotype 8, highly pathogenic avian influenza in poultry, foot-and-mouth disease, and classical swine fever [65,66,67,68,69].

Several other farm-related factors were considered as well in this study. We did not find evidence for differences in the risk of infection, clinical signs, and prevalence on farms between mink farms with closed halls compared to open sheds. Furthermore, similar to analyses of outbreaks on mink farms in other countries, there was no indication that the virus was transmitted through shared service vehicles, materials, feed suppliers, veterinarians, or movement of animals or animal products [12,17,37]. One-third of the infected farms applied slurry on pastures, prior to being diagnosed, near their own and other mink farms. It is not clear whether and how much viable virus this slurry may have contained and whether this could have been transmitted to other mink farms. It is not known how long the SARS-CoV-2 virus can remain infectious in manure [17]. Laboratory tests performed by WBVR (Lelystad, the Netherlands), in which SARS-CoV-2 was added to dry manure and slurry tanks, showed that viral RNA could still be detected after several weeks, although the cell cultures soon tested negative [40]. Based on the distances, time of application, and the time other mink farms became infected, it was considered unlikely that manure application on the land played a role in between-farm spread [40]. Furthermore, no evidence of this transmission route was found in outbreak analyses performed in other countries [12,17].

Many farms had guard dogs on the premises, but domestic cats were only present on a few farms. In contrast, many farms reported that feral cats were frequently roaming around on the farm. On ten of the infected mink farms, 3% of 101 tested feral cats and 8% of 13 dogs tested SARS-CoV-2 PCR positive, and 11 cats (18%) and two dogs (15%) tested serologically positive [13]. Evidence for mink-to-cat transmission but not for cat-to-cat transmission was found. Based on virus sequences of nearby infected farms, the role of cats in the farm-to-farm spread was not considered likely, which was also the case for the Danish outbreaks [13,17]. Other free-roaming animal species, such as birds, wild carnivores, bats, and rodents, were also often reported on the farms. These findings are in line with the conclusions of risk assessments by wildlife and ornithological experts, who indicated that the open housing system on the Dutch mink farms makes it likely for birds, bats, and most free-ranging carnivores to enter and exit mink farms and come into direct contact with mink of infected mink materials. This could potentially lead to infection or mechanical carriage of the virus to other farms [37,41]. Although SARS-CoV-2 RNA was found on the feet of two dead house sparrows (*Passer domesticus*) found on two mink farms and in two escaped mink [37,40], none of the other tested carcasses and fecal and lung samples of several different wild carnivores, bat and bird species tested positive [41]. This makes it unlikely that SARS-CoV-2 circulated abundantly in the wildlife population [40,41]. Nevertheless, considering the small distances between farms, free-roaming animals may have played a role in the farm-to-farm spread in some of the cases.

## 5. Conclusions

In the Netherlands, the continued spread prompted an earlier ban on mink farming. In countries where mink farming continues, the information presented in this study may contribute to improving clinical and diagnostic surveillance and containment measures for SARS-CoV-2 infections in mink. Moreover, it may also be relevant for similar zoonotic infections in the future in the context of public and animal health.

Although contact between infected humans and minks, and vice versa, as introduction and transmission route could explain some SARS-CoV-2 infections on mink farms, other factors explaining the continued farm-to-farm spread have not yet been elucidated. This highlights that infections such as SARS-CoV-2, which affect multiple hosts and can also be spread through the environment, require extensive research and surveillance in a One Health context. Moreover, the exchange of data and communication between official medical authorities and official veterinary authorities within and between countries is also an important recommendation [21]. Furthermore, our study showed that infections in mink could be subclinical at individual and farm level, which can complicate timely diagnosis. Therefore, early warning surveillance with frequent testing of mink is essential to reduce risks of a further spread between mink on the farm and subsequently potential spread to other mink farms, mutations of the virus, and infections jumping back and forth from mink to the human population. Furthermore, strict and continuous adherence to hygiene and biosecurity protocols by farm workers is important, and in addition to vaccination of mink farm staff, parallel vaccination of farmed mink [21,33,70] may also contribute to reducing mink–mink and mink–human transmission, but this may require further study.

## Figures and Tables

**Table 1:** Summary of farm and outbreak characteristics and virus types for the 69 mink farms diagnosed with SARS-CoV-2.

Observations	Yes	No	Unknown	Total
Farm characteristics				
Farm distance (<3 km) ^1^	51	18	0	69
Farm distance with the same virus cluster (<3 km) ^2^	43	25	3	69
Type of housing	Sheds = 39; Halls = 21; Both = 6		3	69
Farm size ^3^	Small = 27; Medium = 18; Big = 24		0	69
Sharing of materials and vehicles	6	57	6	69
Same owner	32	37	0	69
Shed/hall visitors (≤5)	35	34	0	69
Exchange of employees (≥1)	36	29	4	69
Permanent employees (≤2)	44	25	0	69
Seasonal workers (≤3)	54	15	0	69
Temporary employees (≥1)	18	51	0	69
Symptomatic employees ^4^	31	20	18	69
Manure	21	39	9	69
Feed supplier ^5^	A = 45; B = 15; C = 9		0	69
Veterinarian ^6^	A = 54	B = 15	0	69
Cats	9	51	9	69
Dogs	41	25	3	69
Feral cats or neighbor cats	50	17	2	69
Other animals ^7^	27	22	20	69
Wildlife including bats and birds	53	1	15	69
Rodents	18	32	19	69
Insects	21	3	45	69
Outbreak characteristics				
Date of diagnosis ^8^	<August = 27	≥August = 42	0	69
Virus type				
Virus cluster ^9^	A = 41; B = 1; C = 15; D = 7; E = 1		4	69

^1^ Distance from (an)other infected mink farm(s); ^2^ Distance from (an)other infected mink farm(s) with the same virus cluster; ^3^ Small ≤ 30,000, medium = 30,000–50,000, big ≥ 50,000; ^4^ Employees reported with signs related to SARS-CoV-2; ^5^ The different letters (A, B, C) stand for the three different feed suppliers; ^6^ The different letters (A, B) stand for the different veterinary practitioners. The majority of the farms were visited by two veterinarians working for the same company. B stands for the other four veterinary practitioners; ^7^ Cattle, sheep or goats, deer, horses, pigs, poultry, rodents, turtles, foxes and wallabies; ^8^ Before or after 1 August 2020; ^9^ Cluster B and E were only detected at one infected mink farm respectively and were not included in the statistical analyses. For four farms the virus cluster could not be determined [15].

**Table 2:** Summary statistics and odds ratio (OR) and 95% confidence interval (95% CI) for the presence of clinical signs at farm level with virus cluster and time of diagnosis in kits or adult females for all 69 infected farms.

Variable	Categories	N ^1^	Mean ^2^	OR ^3^	95% CI ^3^
Cluster	A	41	5.27	Ref ^3^	
	C vs. A	15	4.00	0.81	0.45–1.45
	D vs. A	7	1.57	0.22	0.08–0.53 *
Cluster	C	15	4.00	Ref	
	A vs. C	41	5.27	1.23	0.69–2.21
	D vs. C	7	1.57	0.27	0.09–0.72 *
Diagnosis kits ^4^	<August	27	1.44	Ref	
	≥August	42	3.57	4.25	1.90–10.39 *
Diagnosis females ^5^	<August	27	2.67	Ref	
	≥August	42	3.95	1.80	0.99–3.35

^1^ Total number of farms for the given variables; ^2^ Mean number of clinical symptoms observed on the farm at time of official sampling; ^3^ Clustered univariable grouped binomial logistic regression model estimates for the unadjusted odds ratio (OR) and lower and upper values for the 95% confidence interval (CI) around the OR for the likelihood of observing a symptom. ^3^ Ref is the category used as reference. ^4^ Total number of clinical signs in kits at time of diagnosis. This also corresponds to an age of kits <12 weeks before 1 August 2020 and >12 weeks from 1 August 2020 onwards; ^5^ Total number of clinical signs in adult females at time of diagnosis; * Significant associations are indicated with an asterisk.

**Table 3:** The percentage of minks with clinical signs on the seven extensively sampled farms, with for each of the variables with a significant association with clinical signs, i.e., coat color, sex/age, and a positive ELISA test result, the odds ratio (OR) and 95% confidence interval (95% CI) based on the univariable logistic regression models.

Independent Variable	Categories	N ^1^	Clinical Signs (%) ^2^	OR ^3^	95%CI ^3^
Coat color ^4^	Dark: wild, brown, MAH	750	11.9	Ref ^3^	
	Light: SBL and SCR	466	4.07	0.35	0.29–0.60 *
	Other: Jag, pearl, black, other	187	2.19	0.41	0.10–1.42
Sex/age ^5^	Juvenile males	388	11.08	Ref ^3^	
	Juvenile females	521	4.99	0.33	0.19–0.60 *
	Adult females	494	8.70	1.10	0.50–1.29
Sex/age ^5^	Juvenile females	521	4.99	Ref ^3^	
	Juvenile males	388	11.08	3.02	1.78–5.25 *
	Adult females	494	8.70	2.64	1.44–4.19 *
ELISA	Negative	1348	7.03	Ref ^3^	
	Positive	55	23.64	2.64	1.24–5.38 *

^1^ Total number of mink for the given variables; ^2^ Percentage of mink with clinical signs; ^3^ Final univariable mixed effect logistic regression model estimates for the adjusted odds ratio (OR) and lower and upper values for the 95% confidence interval (CI) around the OR for the likelihood of presence of clinical signs for the given variables. ^3^ Ref is the category used as reference. ^4^ Dark coat color = wild, brown or mahogany (MAH), light = silver blue (SBL) and silver cross (SCR), other coat colors = jaguar (Jag), pearl, black, others). ^5^ Juveniles are <1 year, adults >1 year of age; * Significant associations based on the 95% CI are indicated with an asterisk.

## Data Availability

Not applicable.

## Supplementary Materials

The following supporting information can be downloaded at: https://www.mdpi.com/article/10.3390/v14081754/s1, Table S1: List of the 19 scored clinical signs; Table S2: PCR test results for each individual farm including the number of swabs and the number and percentage of positive swabs for both rectal and throat swabs; Table S3: The proportion of kits and females with clinical signs; Table S4: Proportion of farms with positive PCR test results for throat and rectal swabs grouped in prevalence classes for farms diagnosed before August and from August onwards; Table S5: Overview of the dataset of the extensively sampled mink farms; Table S6. Mean Ct-values for the RT-PCR of positive samples on the seven extensively sampled farms, with for the mink-related variables with a significant associations with Ct-value, i.e., sex/age, the hazard ratio and 95% confidence interval (95% CI) based on survival analysis.
