# Supplementary material for: Manifestation of SARS-CoV-2 Infections in Mink Related to Host-, Virus- and Farm-Associated Factors, The Netherlands 2020
Source: Viruses. 2022 Aug 11;14(8):1754. doi: 10.3390/v14081754 (PMC9414453; doi:10.3390/v14081754)
Supplement: Supplementary file 1 [file viruses-14-01754-s001.zip › viruses-1824322-supplementary.pdf]

Supporting information

Table S1. List of the 19 scored clinical signs

General signs	Signs of the respiratory tract	Signs of the gastrointestinal tract
Apathy	*Upper respiratory tract*	Diarrhea
Fever	Watery nasal discharge	Bleeding, inflammation or lesions of the gingiva
Weight loss	Hemorrhagic nasal discharge	
Reduced food intake / anorexia	Watery eye effusion / excessive lacrimation	
Reduced water intake	Conjunctivitis	
Reduced fur quality / poor coat condition	Sinusitis	
Increased mortality	Discoloration of the nose	
	Crusts/lesions on the nose	
	Lower respiratory tract	
	Breathing sounds (sneezing/coughing)	
	Tachypnea	
	Accessory breathing	

Table S2. PCR test results for each individual farm including the number of swabs and the number and percentage of positive swabs for both rectal and throat swabs[1,2]

***NB*[1]**	***Throat swabs***	***Throat swabs (+)***	***Throat swabs % (+)***	***Rectal ,***	***Rectal swabs (+)***	***Rectal swabs % (+)***	***NB***	***Throat ,***	***Throat swabs (+)***	***Throat swabs % (+)***	***Rectal swabs***	***Rectal swabs (+)***	***Rectal swabs % (+)***
NB1a	5	5	100%	5	3	60%	NB35	20	12	60%	20	19	95%
NB1b	9	2	22%	20	0	0%	NB36*	20	3	15%	20	20	100%
NB2	5	5	100%	5	1	20%	NB37	20	20	100%	20	1	5%
NB3	32	14	44%	8	4	50%	NB38	20	17	85%	20	0	0%
NB4	4	2	50%	4	2	50%	NB39	20	19	95%	20	2	10%
NB5	20	2	10%	20	0	0%	NB40	22	22	100%	22	3	14%
NB6	20	13	65%	20	3	15%	NB41	22	19	86%	22	2	9%
NB7	20	8	40%	20	3	15%	NB42	20	20	100%	20	1	5%
NB8	21	16	76%	21	5	24%	NB43	20	20	100%	20	4	20%
NB9	20	1	5%	20	2	10%	NB44	20	9	45%	20	0	0%
NB10	20	0	0%	20	8	40%	NB45*	20	17	85%	20	1	5%
NB11	20	13	65%	20	1	5%	NB46	20	16	80%	20	0	0%
NB12	21	19	90%	21	4	19%	NB47	20	20	100%	20	0	0%
NB13	21	7	33%	20	0	0%	NB48	20	20	100%	20	0	0%
NB14	20	7	35%	20	2	10%	NB49*	40	20	100%	40	0	0%
NB15	20	17	85%	20	4	20%	NB50*	20	17	85%	20	6	30%
NB16	20	8	40%	20	2	10%	NB51	20	20	100%	20	1	5%
NB17*	20	18	90%	20	4	20%	NB52*	20	19	95%	20	0	0%
NB18	21	8	38%	20	3	15%	NB53*	20	20	100%	20	0	0%
NB19	20	16	80%	20	0	0%	NB54*	21	21	100%	21	6	29%
NB20	41	6	15%	41	0	0%	NB55	20	20	100%	20	0	0%
NB21	20	14	70%	20	2	10%	NB56*	22	19	86%	22	14	64%
NB22	20	1	5%	20	0	0%	NB57	20	20	100%	20	6	30%
NB23	20	7	35%	20	2	10%	NB58	20	18	90%	20	3	15%
NB24	20	1	5%	20	0	0%	NB59	20	20	100%	20	4	20%
NB25	21	4	19%	21	0	0%	NB60	20	16	80%	20	9	45%
NB26*	20	20	100%	20	14	70%	NB61*	24	23	96%	23	14	61%
NB27	20	18	90%	20	3	15%	NB62*	20	19	95%	20	9	45%
NB28	20	20	100%	20	8	40%	NB63	20	20	100%	20	12	60%
NB29	20	20	100%	20	1	5%	NB64*	20	20	100%	20	0	0%
NB30*	22	1	5%	22	1	5%	NB65	20	20	100%	20	0	100%
NB31	20	20	100%	20	8	40%	NB66	20	0	0%	20	0	0%
NB32	20	20	100%	20	0	0%	NB67	20	20	100%	20	2	10%
NB33*	20	20	100%	20	6	30%	NB68	20	20	100%	20	3	15%
NB34	20	19	95%	20	3	15%							

[1] The farms which were officially sampled multiple times are marked with an asterisk; [2] The farms NB5-NB26 were diagnosed in the months before August. The farms NB27-NB68 were diagnosed from 1 August onwards.

Table S3. The proportion of kits and females with clinical signs.

	Kits	Females
Clinical signs	0.64	0.75
No clinical signs	0.36	0.25

Table S4. Proportion of farms with positive PCR test results for throat and rectal swabs grouped in prevalence classes for farms diagnosed before August and from August onwards.

Type of swab[1]	Prevalence class[2]		
Throat swabs	< 25%	25-75%	>75%
< August	0.30	0.40	0.30
≥ August	0.07	0.05	0.88
Rectal Swabs	< 25%	25-75%	>75%
< August	0.81	0.19	0.00
≥ August	0.67	0.26	0.07

[1] An average of 20 throat and 20 rectal swabs were taken at each farm from minks with clinical signs or from mink in cages with prior observed mortality if present, and otherwise randomly; [2] The specificity of the PCR test is presumed 100% based on Corman et al., 2020[#].

[#] Corman, V.M.; Landt, O.; Kaiser, M.; Molenkamp, R.; Meijer, A.; Chu, D.K.; Bleicker, T.; Brünink, S.; Schneider, J.; Schmidt, M.L.; Mulders, D.G.; Haagmans, B.L.; van der Veer, B.; van den Brink, S.; Wijsman, L.; Goderski, G.; Romette, J.; Ellis, J.; Zambon, M.; Peiris, M.; Goossens, H.; Reusken, C.; Koopmans, M.P.; Drosten, C. Detection of 2019 novel coronavirus (2019-nCoV) by real-time RT-PCR. Euro Surveill 2020, 25, DOI 10.2807/1560-7917.ES.2020.25.3.2000045. Available online: https://www.ncbi.nlm.nih.gov/pmc/articles/PMC6988269/ (accessed on Jun 24, 2022).

Table S5. Overview of the dataset of the extensively sampled mink farms

NB	Analyzed samples N =	Sex N = …[1]	Age N =…[2]	Color N =…[3]	Positive serology N =	PCR pool N=	PCR Indiv N=	Positive PCR	Animals with Signs N =	Type of Signs N =	Type of housing
All	1523	T = 388<br>R = 1015	J = 908<br>O = 495	Jag = 11<br>Pearl = 87<br>SBL= 422<br>SCR = 45<br>Wild = 633<br>Brown = 16<br>Mahogany = 100<br>Black = 88<br>Other = 1	55	780	504		144		
46	130	T = 90*<br>R = 40	J = 40<br>O = 90*	SBL = 64*<br>Wild = 66	1	36	0	100%	1	Unknown	Hall
48	155	T = 81*<br>R = 74*	J = 91*<br>O = 64*	Jag = 9*<br>Pearl = 16<br>SBL= 76*<br>SCR = 28*<br>Wild = 26*	0	34	0	100%	10	Unknown	Hall
50	112	T = 96*<br>R = 16	J = 79<br>O = 21*<br>G = 12	SBL = 18<br>SCR = 5<br>Wild = 89*	9	18	25	100%	3	Unknown	Sheds
52	232	T = 195*<br>R = 37*	J = 140*<br>O = 92*	Brown = 16<br>FY = 1<br>Jag = 2<br>Pearl = 71<br>SBL = 28<br>SCR = 4<br>Wild = 22*<br>Black = 88*	2	19	141	100%	4	General (3)<br>Respiratory (1)	Halls<br>Sheds
53	314	T = 250*<br>R = 64*	J = 107*<br>O = 207*	MAH = 73*<br>SBL = 78*<br>Wild = 163*	39	39	148	100%	55	General (6)<br>Respiratory (50)<br>Gastro-intestinal (1)	Sheds
54	267	T = 187*<br>R = 80*	J = 207 *<br>O = 60 *	MAH = 27<br>SBL = 113*<br>SCR = 4<br>Wild = 123*	1	39	110	100%	22	General (5)<br>Respiratory (16)	Sheds
61	193	T = 116*<br>R = 77*	J = 143*<br>O = 50*	SBL = 45 *<br>SCR = 4 *<br>Wild = 144 *	3	0	40	100%	17	General (2)<br>Respiratory (13)<br>Gastro-intestinal (1)	Hall

[1] T = female, R = male; [2] J = young (< 1 year), O = adult (1 > year), G = gusted; [3] SBL = silver blue, Jag = jaguar, SCR = silver cross, MAH = mahogany; * Presence of clinical signs is indicated with an asterisk.

Table S6. Mean Ct-values for the RT-PCR of positive samples on the seven extensively sampled farms, with for the mink-related variables with a significant associations with Ct-value, i.e. sex/age, the hazard ratio and 95% confidence interval (95% CI) based on survival analysis.

Independent Variable	Categories	N[1]	Mean	HR[2]	95% CI[2]
Sex/age[3]	Juvenile males	388	21.26	Ref[2]	
	Juvenile females	521	22.36	0.75	0.60-0.95*
	Adult females	494	22.34	0.77	0.59-0.99*

[1]Total number of mink for the given variables; [2] HR = Hazard ratio (exponentiated coefficients (exp(coef)) and lower and upper values for the 95% confidence interval (CI) around the HR for the given variables. Ref is the category used as reference. [3] Juveniles are <1 year, adults >1 year or age; [*] Significant associations based on the 95% CI are indicated with an asterisk.
